# (Semi)-Synthetic Fucosylated Chondroitin Sulfate Oligo- and Polysaccharides

**DOI:** 10.3390/md18060293

**Published:** 2020-06-01

**Authors:** Giulia Vessella, Serena Traboni, Antonio Laezza, Alfonso Iadonisi, Emiliano Bedini

**Affiliations:** 1 Department of Chemical Sciences, University of Naples Federico II, Complesso Universitario Monte S. Angelo, via Cintia 4, I-80126 Napoli, Italy; giulia.vessella@unina.it (G.V.); serena.traboni@unina.it (S.T.); iadonisi@unina.it (A.I.); 2 Department of Sciences, University of Basilicata, viale dell’Ateneo Lucano 10, I-85100 Potenza, Italy; antonio.laezza@unibas.it

**Keywords:** carbohydrates, polysaccharides, semi-synthesis, sulfation, glycosylation, fucose, fucosylated chondroitin sulfate

## Abstract

Fucosylated chondroitin sulfate (fCS) is a glycosaminoglycan (GAG) polysaccharide with a unique structure, displaying a backbone composed of alternating *N*-acetyl-d-galactosamine (GalNAc) and d-glucuronic acid (GlcA) units on which l-fucose (Fuc) branches are installed. fCS shows several potential biomedical applications, with the anticoagulant activity standing as the most promising and widely investigated one. Natural fCS polysaccharides extracted from marine organisms (*Echinoidea*, *Holothuroidea*) present some advantages over a largely employed antithrombotic drug such as heparin, but some adverse effects as well as a frequently found structural heterogeneity hamper its development as a new drug. To circumvent these drawbacks, several efforts have been made in the last decade to obtain synthetic and semi-synthetic fCS oligosaccharides and low molecular weight polysaccharides. In this Review we have for the first time collected these reports together, dividing them in two topics: (i) total syntheses of fCS oligosaccharides and (ii) semi-synthetic approaches to fCS oligosaccharides and low molecular weight polysaccharides as well as glycoclusters displaying multiple copies of fCS species.

## 1. Introduction

Glycosaminoglycans (GAGs) are highly negatively charged polysaccharides ubiquitously distributed in the animal kingdom. They are usually found covalently linked to proteins to form proteoglycans (PGs), which are one of the major and most important components of the extracellular matrix. GAGs are involved in a myriad of biological events in both physiological and pathological processes [1]. From a structural point of view GAG polysaccharides are constituted of a linear sequence of disaccharide units, each consisting of an aminosugar and a hexose or an uronic acid, very often decorated with one or more sulfate groups on their structure. 

Some marine invertebrates display GAGs with unique, non-linear structures, characterized by the presence of monosaccharide or short oligosaccharides as branches [2,3]. Among these branched GAGs, most attention is currently focused on fCS, a glycosaminoglycan found up to now exclusively in the body wall of sea cucumbers (*Echinoidea*, *Holothuroidea*) and showing several potential biomedical applications related to inflammation, hyperglycemia, atherosclerosis, cellular growth, cancer metastasis, and angiogenesis [4]. However, its most interesting feature is the potential use as an antithrombotic agent alternative to heparin [5], compared to which fCS displays two very important advantages: (i) unlike heparin, the activity is retained also on antithrombin (AT)- and heparin cofactor II (HC-II)-free plasmas, because the mechanism of action of fCS on the blood coagulation cascade has some differences with respect to heparin [6,7]; (ii) it is orally deliverable because it can be digested neither during its adsorption in the gastrointestinal tract nor by intestinal bacterial enzymes [8]. 

**Figure 1:**
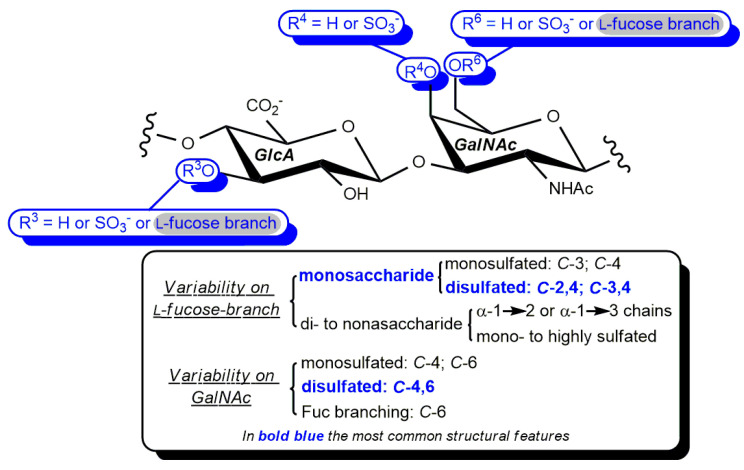
Structural variability of fCS from sea cucumbers.

The unique structure of fCS consists of a linear backbone of GalNAc and GlcA units linked together through alternating β-1→3 and β-1→4 glycosidic bonds, on which a single Fuc unit or, more rarely, di- to nonasaccharide Fuc chains are inserted as branches. Sulfate groups are also present to a various extent both on the backbone and on Fuc branches [9]. The latter, which are essential for the biological activities of fCS [10,11,12], are usually linked at position *O*-3 of GlcA residues, even if different fucosylation sites have been sometimes found too [13,14,15,16]. The structural diversity of fCS from different sea cucumbers species is mainly investigated by two-dimensional nuclear magnetic resonance (2D–NMR) techniques [17] and achievements in this field have been reviewed [9,18,19]. Since the structural elucidation of fCS polysaccharides from more and more species is constantly published, upgraded summarizing tables can be found in very recent papers [20,21]. A schematic representation of fCS structural variability found up to now is depicted in Figure 1.

Although fCSs have shown very interesting anticoagulant and antithrombotic activities, the intravenous administration of the native polysaccharides can cause some adverse effects such as platelet aggregation, hypotension, and bleeding [22]. These drawbacks are typically associated with high molecular weight (M_w_) sulfated species. Indeed, M_w_ values of fCS polysaccharides typically span from 20 to 100 kDa [23]. To develop fCS-based anticoagulant and antithrombotic lead compounds while avoiding these side effects, in the last decade several research groups have focused their attention on the production of fCS oligosaccharides and low molecular weight polysaccharides through total synthetic or semi-synthetic approaches. This has also helped detailed structure-activity relationship studies that are instead hampered by the usually heterogeneous structure of native fCS polysaccharides. Indeed, only in a few cases a homogeneous structure with a single sulfation pattern on Fuc and GalNAc units has been found [21,24,25,26]. Even if the synthesis of GAG oligo- and polysaccharides has been recently reviewed [27,28,29], to the best of our knowledge no comprehensive account selectively focusing on the synthetic and semi-synthetic efforts to fCS species that increased more and more in the last decade, has been published. Here we fill this gap, including in this review also the very recent reports on the obtainment of fCS-based multivalent structures. The content is divided into two chapters: the former reviews the total synthetic approaches for building fCS oligosaccharides starting from commercially available monosaccharides, the latter concerns the semi-synthetic strategies to obtain fCS oligosaccharides and low molecular weight polysaccharides starting from natural fCS itself or related GAGs.

## 2. Total Synthetic Approaches

**Scheme 1:**
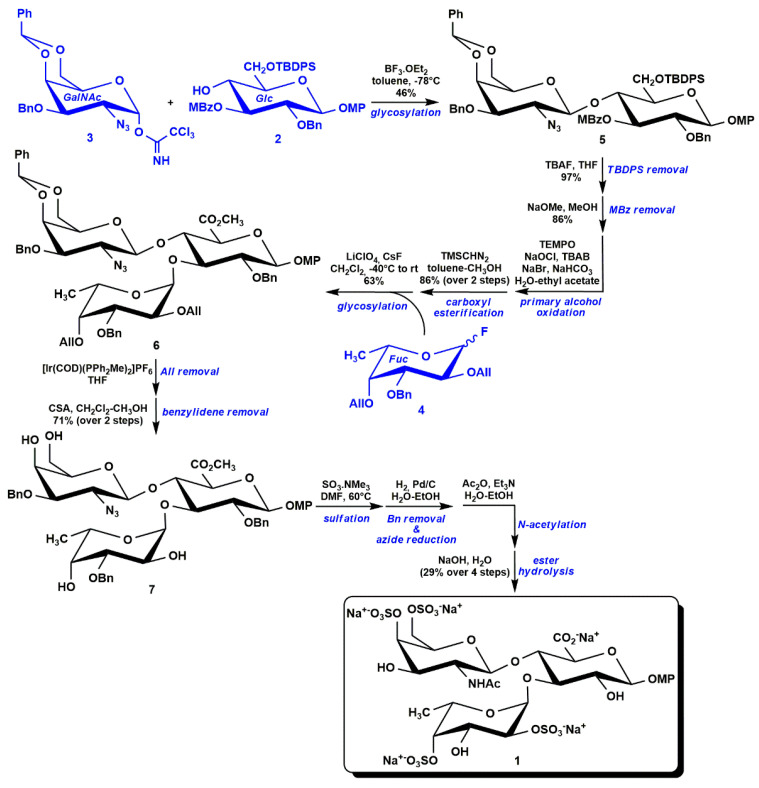
First synthesis of fCS trisaccharide by Tamura and co-workers [30]; abbreviations: acetic anhydride (Ac_2_O), (1*S*)-(+)-10-camphorsulfonic acid (CSA), 1,5-cyclooctadiene (COD), benzyl (Bn), *p*-methoxyphenyl (MP), tetra-*n*-butylammonium bromide (TBAB), tetra-*n*-butylammonium fluoride (TBAF), 2,2,6,6-tetramethyl-1-piperidinyloxy free-radical (TEMPO), tetrahydrofuran (THF), (trimethylsilyl)diazomethane (TMSCHN_2_).

The first total synthesis of a fCS oligosaccharide was reported by Tamura and co-workers in 2013 [30]. They synthesized the trisaccharide unit most commonly found in natural fCS―4,6-di-*O*-sulfated-β-GalNAc-(1→4)-[2,4-di-*O*-sulfated-α-Fuc-(1→3)]-GlcA―as β-*p*-methoxyphenyl glycoside (**1**, Scheme 1), starting from monosaccharide building blocks **2**–**4** that were carefully designed with respect to their orthogonal pattern of protecting groups. 

In particular, glucose (Glc) derivative **2** displayed a free alcohol at *C*-4 site together with a *p*-methoxybenzoyl (MBz) ester and *t*-butyldiphenylsilyl (TBDPS) ether installed at *C*-3 and *C*-6 position, respectively. These two protecting groups could be orthogonally cleaved to liberate the corresponding hydroxyls that could be then fucosylated or oxidized at the desired synthetic stage. Similarly, trichloroacetimidate donor **3** served as building block for the GalNAc unit, with an azide as acetamide masking group and a benzylidene as orthogonally cleavable protecting group for *C*-4,6 diol, which had to be sulfated at a late stage of the synthesis. Finally, glycosyl fluoride **4** was chosen as Fuc donor, displaying allyl (All) ethers as temporary protecting groups at *C*-2 and *C*-4 positions that should be sulfated as well. The assembly of the building blocks started with the glycosylation between acceptor **2** and donor **3** to give disaccharide **5**, that after TBDPS and MBz cleavage, primary alcohol oxidation, carboxyl esterification and α-fucosylation, afforded the fully protected trisaccharide **6** in 21% overall yield. Then, selective removal of allyl and benzylidene protecting groups liberated the alcohol moieties at the positions selected for sulfation that was conducted with SO_3_·NMe_3_ complex in *N*,*N*-dimethylformamide (DMF) before full deprotection in three steps to afford the target fCS trisaccharide **1** with a 4.3% global yield, calculated from the Glc, GalNAc and Fuc monosaccharide building blocks.

**Scheme 2:**
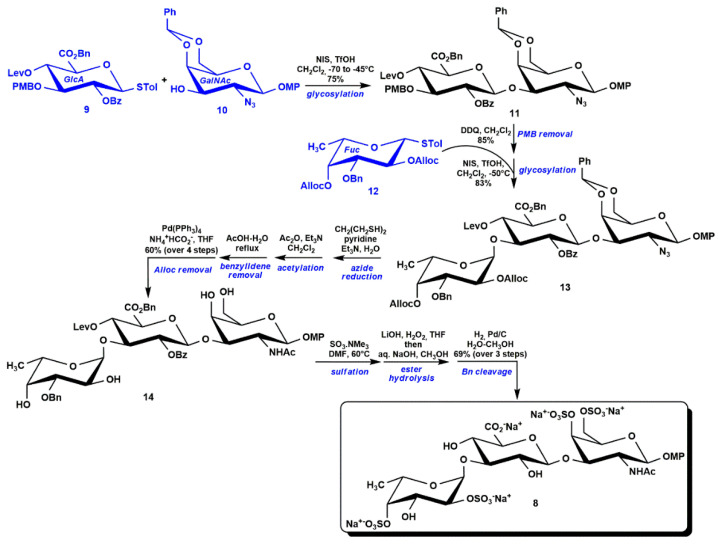
Synthesis of fCS trisaccharide with GalNAc unit at pseudo-reducing position by Qi and co-workers [31]; abbreviations: benzoate (Bz), 2,3-dichloro-5,6-dicyano-*p*-benzoquinone (DDQ), levulinoyl (Lev), *N*-iodosuccinimide (NIS), trifluoromethanesulfonic acid (TfOH), toluene (Tol).

Most recently, Qin and co-workers reported the total synthesis of fCS trisaccharide **8**, displaying the same sulfation pattern as **1** but with the GalNAc unit instead of GlcA at the pseudo-reducing position [31] (Scheme 2). 

In their synthetic strategy the *C*-6 oxidation on Glc to give a GlcA unit was performed at a monosaccharide level, therefore the first glycosylation was conducted between the suitably protected GlcA thioglycoside donor **9**, carrying a *p*-methoxybenzyl (PMB) ether as orthogonally cleavable protecting group at *C*-3 site, and GalNAc acceptor **10**, again with an azide as acetamide masking group (also several *N*-protecting groups were tested but with much worse results). The obtained disaccharide **11** was subjected to a selective cleavage of the PMB ether to liberate a single hydroxyl that was in turn glycosylated in the presence of Fuc thioglycoside donor **12**, carrying an allyloxycarbonyl (Alloc) as orthogonal protecting group at *C*-2 and *C*-4 positions. Interestingly, an α-linked glycosidic bond was formed, although Alloc protecting group at *C*-2 site would direct to a 1,2-*trans* β-stereochemistry due to a neighboring participating effect in the glycosylation mechanism. The authors suggested that the obtainment of the 1,2-*cis* α-product was due to an in situ anomerization from the firstly formed β-trisaccharide to the more stable α-anomer. Fully protected trisaccharide **13** was then subjected to azide-acetamide conversion and the alcohol moieties were liberated at the positions selected for sulfation by Alloc and benzylidene cleavage. Tetraol **14** was sulfated and then globally deprotected under conditions similar to the synthesis of **1**. Target fCS trisaccharide **8** was obtained in 26% overall yield from the GlcA, GalNAc and Fuc monosaccharide building blocks.

Nifantiev and co-workers reported the synthesis and conformational analysis of a library of di- and trisaccharide fragments carrying Fuc branches differing by length, sulfation pattern and site of linkage [32,33,34]. These serve as simple model compounds covering the structural variability of native fCS, including the less frequent cases of Fuc units linked at GalNAc *O*-6 instead of GlcA *O*-3 position [13,14,15,16] and of Fuc oligosaccharides instead of monosaccharides as branches [21,35,36].

**Scheme 3:**
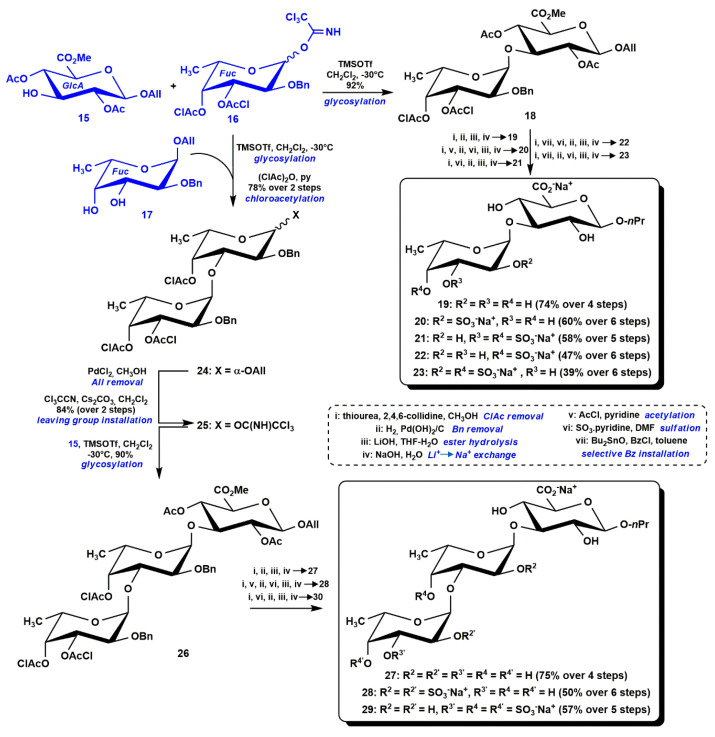
Nifantiev synthesis of di- and trisaccharides with Fuc branches at GlcA *O*-3 site [32]; abbreviations: acetyl chloride (AcCl), benzoyl chloride (BzCl), chloroacetic anhydride ((ClAc)_2_O), *n*-propyl (*n*Pr), trimethylsilyl trifluoromethanesulfonate (TMSOTf).

**Scheme 4:**
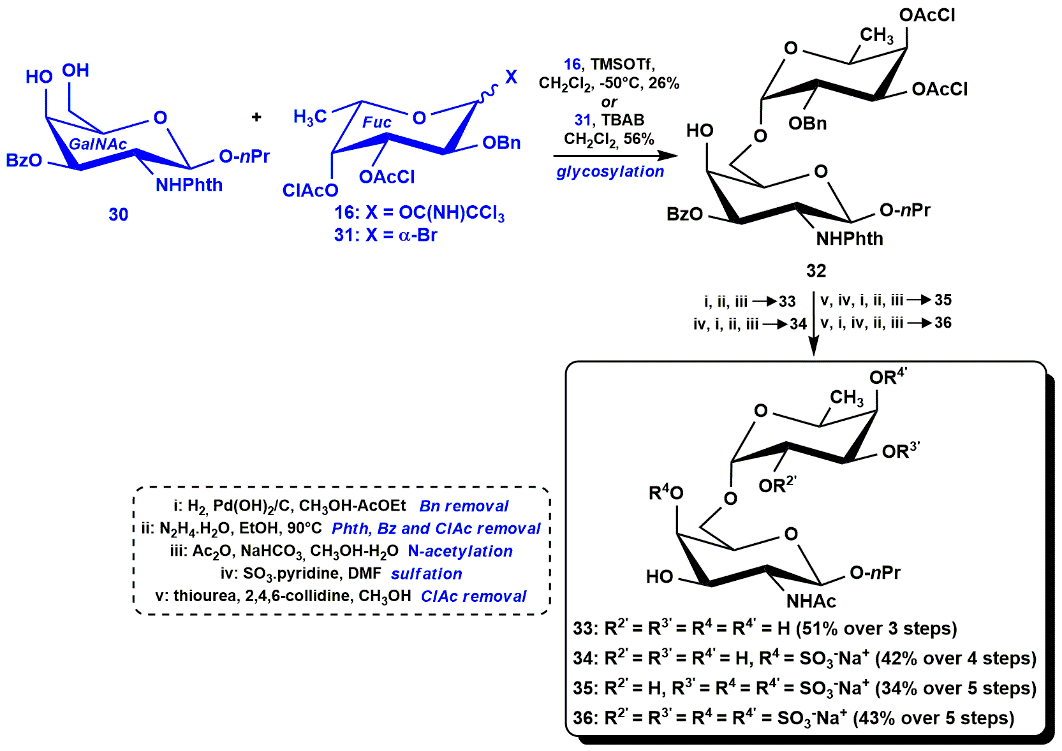
Nifantiev synthesis of disaccharides with Fuc branches at GalNAc *O*-6 site [34]; abbreviations: ethyl acetate (AcOEt), phthaloyl (Phth).

A divergent approach was employed to afford 12 different oligosaccharides from only four monosaccharide building blocks (**15**–**17** and **30**, Scheme 3 and Scheme 4) that were designed with a suitable pattern of permanent and orthogonally cleavable protecting groups. In particular, GlcA acceptor **15** and Fuc trichloroacetimidate donor **16** and acceptor **17** were used to prepare five disaccharides and three trisaccharides (**19**–**23** and **27**–**29**, respectively, Scheme 3), all displaying Fuc branches at GlcA *O*-3 site and differentiated for the length of the branch and/or the sulfation pattern [32]. The five disaccharides were all obtained from the completely protected precursor **18** that was prepared in turn by a glycosylation reaction between **15** and **16**. The very high yield in the α-anomer was ascribed to a remote participation of the chloroacetyl (ClAc) groups at Fuc donor *O*-3 and *O*-4 positions, with the formation of a stabilized glycosyl cation during the glycosylation mechanism. Then, different sequences of few synthetic steps on **18**, including orthogonal protecting group – ClAc or Bn – cleavage, regioselective installation of Bz ester protecting group, sulfation and global deprotection, allowed the obtainment of differently sulfated disaccharides **19**–**23**.

The access to trisaccharides **27**–**29** required firstly the synthesis of Fuc-Fuc disaccharide **24** by a regio- and α-stereoselective glycosylation between donor **16** and acceptor **17**. Further manipulation of **24** by chloroacetylation, de-*O*-allylation and installation of a trichloroacetimidate leaving group at the anomeric position furnished disaccharide donor **25** that was in turn glycosylated with GlcA acceptor **15**. The obtained trisaccharide **26** was finally subjected to three different reaction sequences to afford differently sulfated trisaccharides **27**–**29**.

The synthesis of four disaccharides with Fuc branch linked at GalNAc *O*-6 site was accomplished by using GalNAc acceptor **30** and a Fuc donor [34]. Interestingly, Fuc trichloroacetimidate **16** gave in this case a moderate yield (51%) and no stereoselectivity (α/β 1:1), whereas Fuc bromide **31** afforded α-linked disaccharide **32** exclusively (Scheme 4), even if a much longer reaction time (7 days vs. 30 min) was necessary. As with di- and trisaccharides of Scheme 3, proper sequences of further synthetic steps furnished differently sulfated disaccharides **33**–**36** (Scheme 4).

## 3. Semi-Synthetic Strategies

### 3.1. Semi-Synthesis of fCS Oligosaccharides

**Scheme 5:**
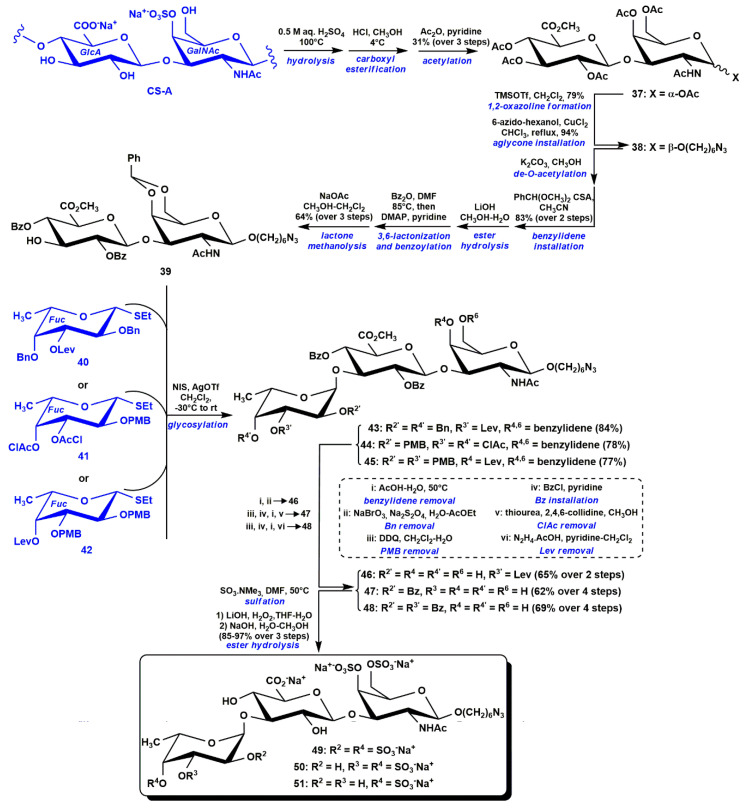
Semi-synthesis of three fCS trisaccharides from CS-A [40]; abbreviations: silver trifluoromethanesulfonate (AgOTf), benzoic anhydride (Bz_2_O).

In addition to the total synthesis of fCS oligosaccharides, the research on fCS species production has concomitantly developed also semi-synthetic strategies based on GAGs or similar natural polysaccharides as starting material. Since chondroitin sulfate (CS) and fCS have very similar molecular structures, the former sharing the same polysaccharide backbone with the latter but without any Fuc branch on it, the use of CS polysaccharides, ubiquitously widespread in the animal kingdom, as starting material for the access to fCS species was pursued. Fifteen years ago, it was already demonstrated that a selective hydrolytic breakage of β-1→4 vs. β-1→3 glycosidic linkages in CS polysaccharides can be carried out under controlled acid conditions, thus obtaining the β-GlcA-(1→3)-GalNAc disaccharide in multi-decagram quantities and in a much shorter time than with any known total synthesis [37,38,39]. Very recently, this approach has been employed for the obtainment of fCS trisaccharides and glycoclusters derived from them [40,41]. By adding two further steps after the controlled acid hydrolysis, peracylated disaccharide **37** could be obtained in 31% yield over three steps from commercially available CS-A polysaccharide (Scheme 5) [40]. It was then subjected to a two-step reaction sequence for the insertion of an aglycone carrying an azide moiety useful for fCS glycocluster synthesis. The obtained disaccharide **38** was further derivatized in order to liberate a single hydroxyl at GlcA *C*-3 site, where Fuc branch had to be attached. This was accomplished by de-*O*-acetylation followed by installation of a benzylidene ring on GalNAc 4,6-diol and of Bz esters at GlcA 2,4-hydroxyls. The latter regioselective reaction was possible through a one-pot 3,6-lactonization/benzoylation/lactone methanolysis sequence [42]. Disaccharide acceptor **39** was obtained in 39% overall yield over eight steps from **37**. Its coupling with three differently protected Fuc thioglycoside donors **40**–**42** gave trisaccharides **43**–**45** in high yield and α-stereoselectivity, as expected for the remote participation of Lev or ClAc esters protecting position *O*-3 and/or *O*-4 of Fuc donors. Fully protected derivatives **43**–**45** were then subjected to different sequences of orthogonal protecting groups cleavage to give trisaccharides **46**–**48** with some liberated hydroxyls that could be in turn sulfated and subjected to a final global ester hydrolysis to afford target fCS trisaccharides **49**–**51** carrying a 2,4-*O*-, 3,4-*O*- or 4-*O*-sulfate decoration, respectively, on Fuc unit.

**Figure 2:**
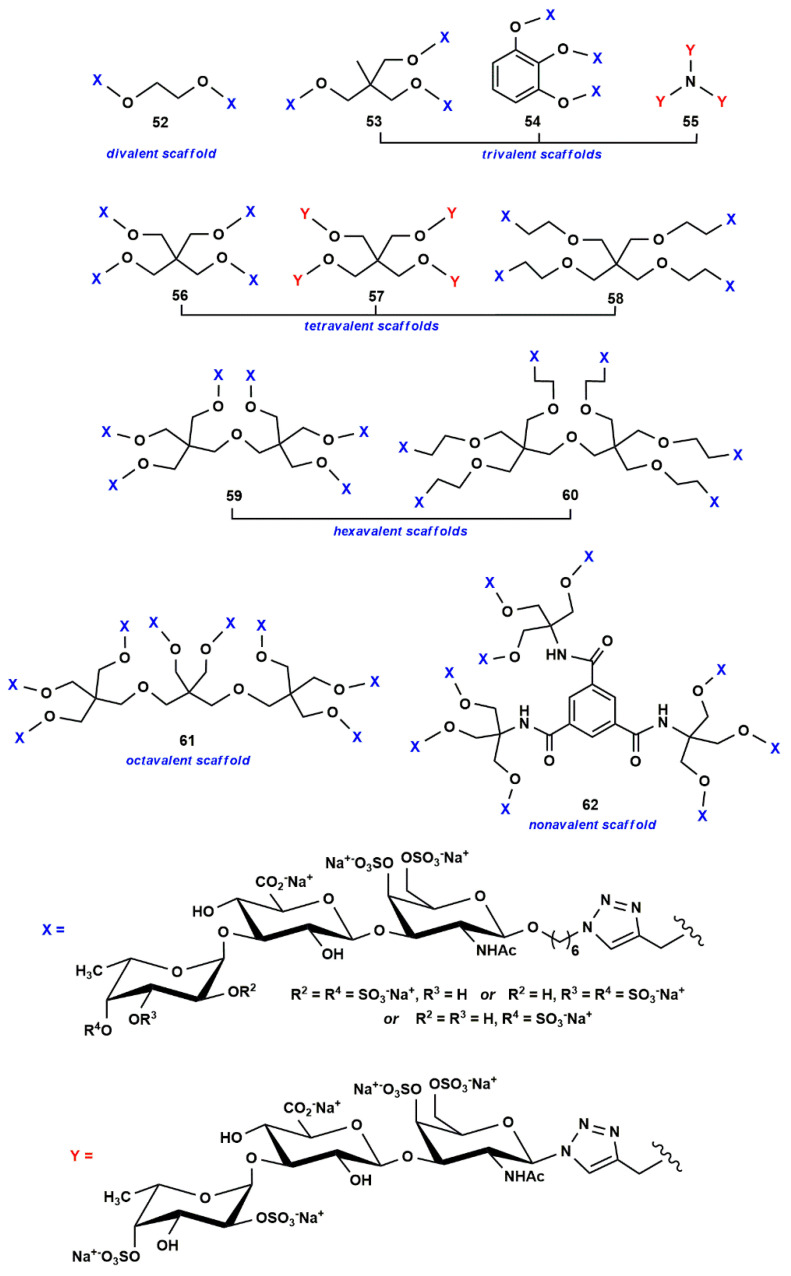
fCS glycoclusters semi-synthesized from CS-A [40,41]

The three partially protected, semi-synthetic trisaccharides **46**–**48** were also employed for the construction of a library of fCS glycoclusters displaying three different Fuc sulfation pattern and nine different multivalent scaffold architectures (**52**–**54**, **56**, **58**–**62**, Figure 2). The multiple decoration of the scaffolds with the fCS trisaccharides was accomplished by exploiting the azide moiety of **46**–**48** aglycone in a Cu(I)-catalyzed azide-alkyne cycloaddition (CuAAC) “click” reaction in a ternary solvent mixture (CHCl_3_–CH_3_OH–H_2_O), followed by sulfation and ester hydrolysis under the conditions already employed for **49**–**51**.

Two additional glycoclusters **55** and **57**, carrying a shorter linker between the 2,4-di-*O*-sulfated fCS trisaccharide moieties and the core scaffolds, were reported too. A very similar semi-synthetic approach from CS-A was employed in this case, with the only relevant difference lying in conducting the CuAAC reactions directly on the final fCS trisaccharide under aqueous conditions that were suitably optimized to avoid the interference of the anionic sulfates with the copper catalyst [41]. The activity of glycoclusters **52**–**62** in blocking the intrinsic, extrinsic, and common coagulation cascade pathways was evaluated by measuring the activated partial thromboplastin time (APTT), prothrombin time (PT) and thrombine time (TT), respectively. Hexa-, octa- and nonavalent scaffolds all showed a significant intrinsic pathway inhibition. The octavalent glycocluster **61** carrying 2,4-*O*-disulfated Fuc moieties was the most active one [40], with an APTT value only one order of magnitude higher than natural fCS polysaccharide from *Stichopus monotuberculatus* possessing 92% of Fuc units with the same sulfation pattern [43]. Interestingly, “long-armed” glycoclusters **60** showed a significantly lower inhibition activity than “short-armed” **59**, although the two scaffolds are both hexavalent. It is also worth noting that trivalent and tetravalent glycoclusters **55** and **57**, displaying a shorter linker connecting the fCS trisaccharide to the core moiety, gave an intrinsic pathway inhibition activity comparable or even higher than hexa-, octa- and nonavalent glycoclusters **59**–**62** designed with a longer linker between the oligosaccharides and the core region [41]. These findings clearly indicated that the arrangement of the fCS repeating units in the glycoclusters makes great difference on the bioactivity. 

**Scheme 6:**
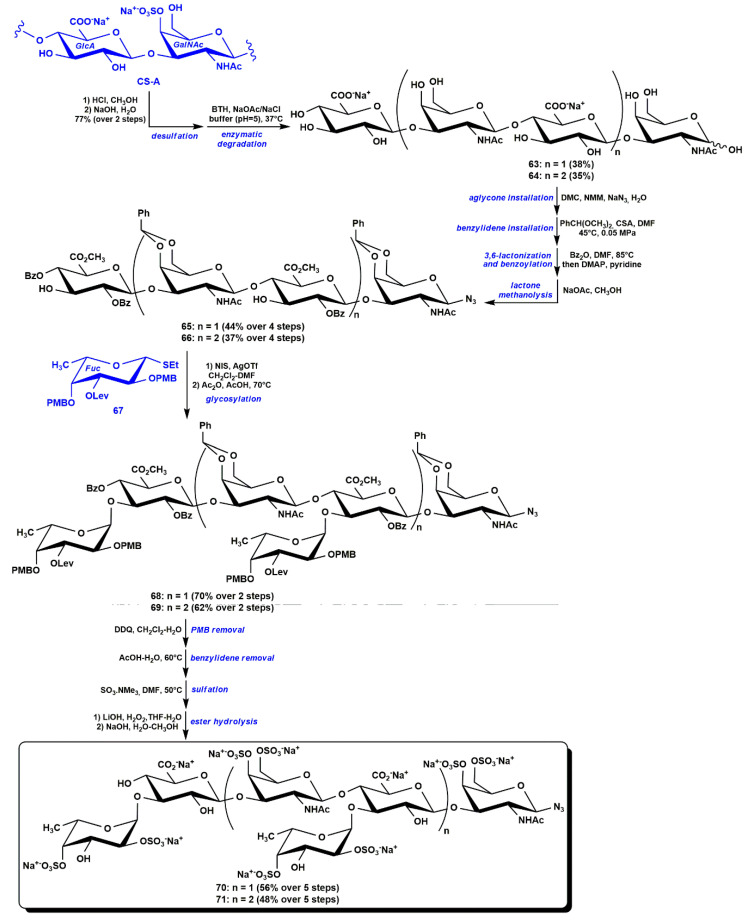
Semi-synthesis of fCS hexa- and nonasaccharide from CS-A [42]; abbreviations: 4-(dimethylamino)pyridine (DMAP), sodium acetate (NaOAc).

**Scheme 7:**
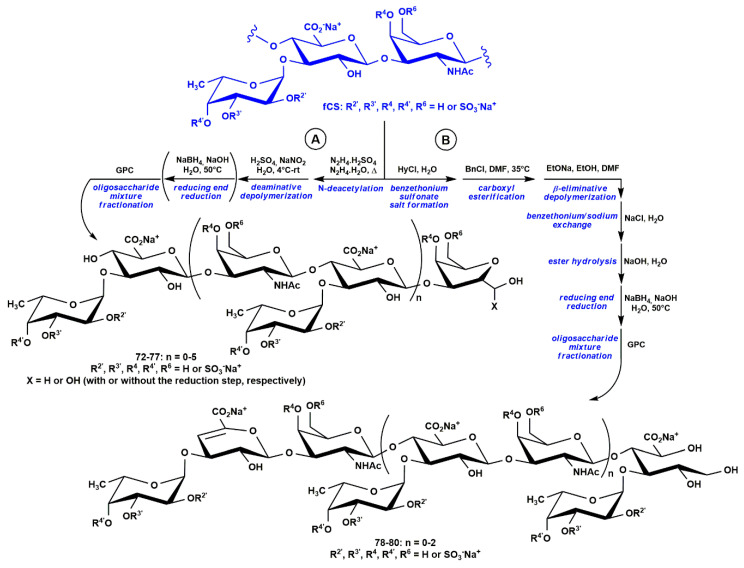
Selective depolymerization (A: deamination; B: β-elimination) protocols for the obtainment of fCS oligosaccharides from natural fCS polysaccharides; abbreviations: benzyl chloride (BnCl), sodium ethoxide (EtONa), benzethonium chloride (HyCl).

Natural CS polysaccharides were employed for the access to fCS species not only as starting material of β-GlcA-(1→3)-GalNAc disaccharide through a controlled chemical hydrolysis, but also for the production of longer oligosaccharides by enzymatic degradation [44]. Indeed, bovine testicular hyaluronidase (BTH) is known to catalyze the breakage of hyaluronic acid and chondroitin polysaccharide chains to the respective tetra- and hexasaccharide species [45]. A decagram scale BTH-catalyzed degradation of desulfated CS afforded [β-GlcA-(1→3)-GalNAc]_2_ tetrasaccharide **63** and [β-GlcA-(1→3)-GalNAc]_3_ hexasaccharide **64** in 38% and 35% yields, respectively (Scheme 6) [42]. On the two oligosaccharides, an azide aglycone was firstly installed selectively at the reducing anomeric position with sodium azide and *N*-methylmorpholine (NMM) in the presence of Shoda reagent (2-chloro-*N*,*N*′-1,3-dimethylimidazolium chloride, DMC) [46]. Then, in order to protect all the functionalities but the hydroxyls at *O*-3 position of GlcA units, a sequence of protection and deprotection steps following the already reported manipulation of disaccharide **38** into **39** (Scheme 5) was demonstrated to be efficient also on tetra- and hexasaccharides. With oligosaccharide acceptors **65** and **66** in hands, challenging double and triple fucosylations with suitably protected Fuc thioglycoside **67** were attempted (Scheme 6). Optimized conditions included a post-glycosylation treatment with Ac_2_O in hot acetic acid to rearrange glycosyl imidate byproducts, formed by acetamide competition as nucleophile [47], into the desired hexa- and nonasaccharide **68** and **69** in 70% and 62% yield, calculated from tetra- and hexasaccharide acceptors **65** and **66**, respectively. Final steps of the synthesis were the cleavage of PMB and benzylidene protecting groups to liberate hydroxyls at Fuc *O*-2,4 and GalNAc *O*-4,6 sites that were then sulfated, and the hydrolysis of the ester protecting groups. Target fCS hexa- and nonasaccharide **70** and **71** were obtained in 5.0% and 3.0% global yield, respectively, from CS-A. It is worth noting that only nonasaccharide **71** showed a significant intrinsic pathway anticoagulation activity, in agreement with the concomitant finding of fCS octasaccharide **79** (see Scheme 7 and discussion below) as the minimum structural unit able to confer anticoagulant activity [48]. Nonasaccharide **71** was also further reacted with a biotin-appended alkyne in a CuAAC reaction involving azide aglycone. The obtained glycoconjugate and a soluble human factor IXa were found by biolayer interferometry studies to fit well to a 2:1 binding model [44].

With the aim to avoid too many chemical reactions on complex oligosaccharides, including the challenging multiple fucosylations discussed above, an alternative approach employing natural fCS polysaccharides as starting material of controlled, partial depolymerizations to fCS oligosaccharides was widely pursued. Several different degradation methods have been investigated. Mild acid hydrolysis afforded a selective cleavage of Fuc branches together with sulfate groups, thus resulting in a valuable method for unveiling the details of natural fCS structures through a bottom-up approach [23,49,50,51] but not applicable for the production of pure fCS oligosaccharides. fCS backbone depolymerizations without a significant loss of sulfate and Fuc branches were reported under hydrothermal [52], ^60^Co γ-rays irradiation [53] and free-radical oxidative conditions. The last method typically employs a Cu(II)-catalyzed Fenton system in a H_2_O_2_ aqueous solution [54,55,56,57], and some careful studies of the influence of several reaction parameters on the rate and extent of depolymerization have been reported [58,59]. Nonetheless, all these methods resulted in no or only slight selectivity in the breakage of GlcA vs. GalNAc glycosidic bonds (or *vice versa*) of the backbone, thus giving very complex mixtures of fCS oligosaccharides for which no fractionation to pure species was reported but in a single report [60].

Instead, this was possible by applying two different multi-step protocols for the highly selective breakage of the glycosidic linkage at the anomeric site of only GalNAc units. One method is based on a deaminative cleavage with nitrous acid, as developed three decades ago firstly for heparin and then for other GAGs [61]. Firstly, fCS was subjected to hydrazinolysis to have a partially de-*N*-acetylated polysaccharide. The degree of de-*N*-acetylation could be varied from 1% to 78% in dependence of several reaction parameters [62]. A subsequent treatment with diluted nitrous acid gave a very fast, highly selective cleavage at *N*-deacetylated GalNAc sites through diazotization of the free amine moieties, and furnished fCS fragments with an unnatural 2,5-anhydro-d-talose unit as reducing end (**72**–**77**, Scheme 7). By modulating the degree of de-*N*-acetylation, fCS oligosaccharides with different length distributions could be obtained in high, overall mass yield (71–80%) [63,64] and then purified by gel-permeation chromatography (GPC) techniques. Pure tri-, hexa-, nona-, dodeca-, pentadeca- and octadecasaccharides with different sulfation patterns have been obtained up to now through this protocol from fCSs extracted from seven different sea cucumber species [62,63,64,65,66]. Among these fCS fragments, nonasaccharide **74** with 2,4-disulfated Fuc branches on GlcA units, 4,6-disulfated GalNAc residues and a 2,5-anhydro-d-talitol as pseudoreducing end revealed to be the minimum fragment retaining the potent selective inhibition of the intrinsic coagulation pathway shown by natural fCS polysaccharide but displaying no side effects [63]. 

A second protocol for the selective cleavage of fCS backbone employs the well-known β-eliminative degradation for polysaccharides containing uronic acid residues [67]. In particular, the method optimized for fCS polysaccharides relies upon a six-step procedure with the key depolymerization step performed on a fCS benzethonium salt derivative with some of the GlcA residues converted into benzyl esters. By treatment of this polysaccharide derivative with sodium ethoxide as base promoting the β-elimination, the breakage of some of the β-1→4 glycosidic linkages involving GlcA esters could be observed (Scheme 7) [68]. Analogously to the hydrazinolysis-deamination protocol discussed above, the modulation of the degree of GlcA esterification allowed the obtainment of fCS fragments with different length distributions in 48-65% overall mass yield [24,25,48]. The obtained mixture could be then fractioned by GPC to furnish pure fCS oligosaccharides. Penta-, octa- and undecasaccharides with different sulfation patterns (**78**–**80**, Scheme 7) were obtained in pure form from fCSs extracted from seven different sea cucumber species [25,26,48,68,69]. Interestingly, these fCS oligosaccharides showed a different structure with respect to those obtained by depolymerization under deamination conditions. Indeed, it is well-known that a peeling reaction can easily occur under alkaline conditions on a 1→3-linked reducing end [70], such as the GalNAc residue released after fCS β-elimination. This resulted in fCS oligosaccharides with a single GalNAc unit missing with respect to oligosaccharides obtained by deamination, and a glucuronitol residue instead of 2,5-anhydro-d-talitol as pseudo-reducing end (Scheme 7). Noteworthy, in this group of fCS oligosaccharides, octasaccharide **79** revealed to be the minimum fragment showing a significant activity as intrinsic coagulation pathway inhibitor [48].

### 3.2. Semi-Synthesis of Low Molecular Weight fCS Polysaccharides

**Scheme 8:**
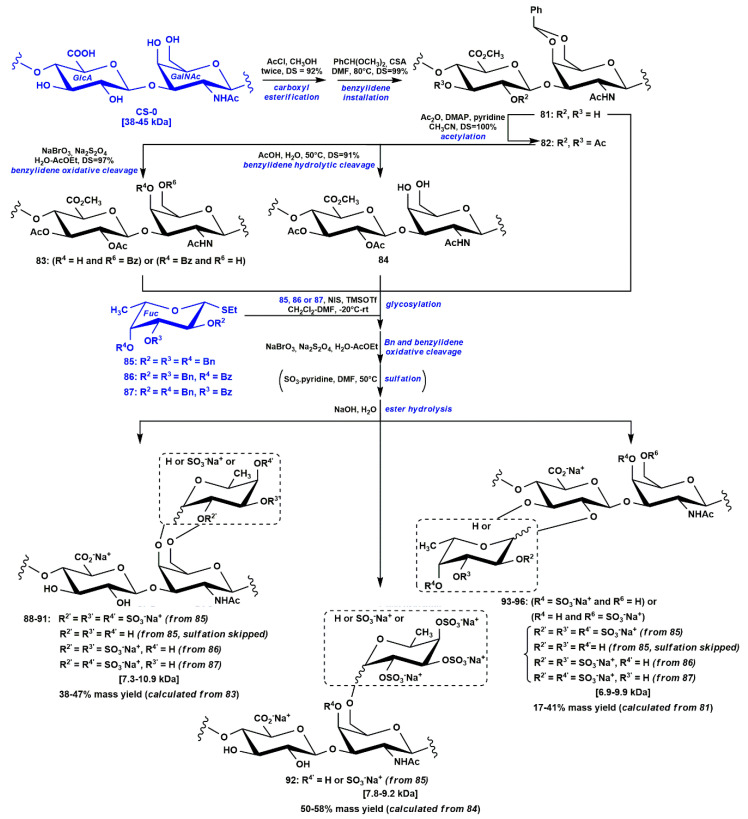
Semi-synthesis of low molecular weight fCS polysaccharides from *E. coli* sourced CS-0.

Semi-synthetic approaches were pursued for accessing not only fCS oligosaccharides but also low molecular weight polysaccharides resembling the structure of natural fCS. Most of these works employed unsulfated chondroitin (CS-0) as starting material that could be obtained by fed-batch fermentation of *Escherichia coli* O5:K4:H4, followed by an expedite downstream purification [71]. This polysaccharide shares with fCS the same polymeric backbone, but is devoid of any decoration of sulfate groups and Fuc branches that could be installed through suitably developed, semi-synthetic sequences [72,73,74]. Indeed, a small library of fCS polysaccharides with different Fuc branching and/or sulfation pattern was obtained [73], by combining two chondroitin polysaccharide acceptors (**81** and **83**, Scheme 8) with three differently protected Fuc donors in challenging glycosylation reactions conducted in a CH_2_Cl_2_-DMF solvent mixture to ensure a high α-stereoselectivity [75]. In particular, chondroitin acceptor **81** resulted from carboxyl esterification followed by protection of GalNAc diol of CS-0, with a nearly quantitative degree of substitution (DS) in both steps. By GlcA diol acetylation and subsequent oxidative cleavage of the benzylidene protecting group [76] on GalNAc units, polysaccharide acceptor **83** could be obtained. Fucosylation of both acceptors was conducted with differently protected donors **85**–**87**. In particular, after a preliminary screening, thioglycosides were selected as better donors than *N*-phenyl-trifluoroacetimidates for both a longer shelf life and a shorter sequence of steps for their preparation, in spite the latter are known to be highly performing in fucosylations [77] and actually gave comparable results to thioglycosides [72]. Fuc donors **85**–**87** displayed benzyl ethers and benzoyl esters as temporary and permanent protecting groups, respectively. Indeed, benzyl ethers could be orthogonally cleaved under oxidative conditions to liberate hydroxyls at specific positions of Fuc units that were then sulfated. This allowed the obtainment of six differently sulfated and/or fucosylated fCS polysaccharides (**88**, **90**, **91**, **93**, **95**, **96**, Scheme 8) with a lower molecular weight (6.9–10.9 kDa) with respect to natural fCS species (20–100 kDa, [23]). Furthermore, by skipping the sulfation step, the two unprecedented non-sulfated, fucosylated chondroitin polysaccharides **89** and **94** could be accessed. Preliminary in vitro anticoagulant assays on the semi-synthesized low molecular weight fCS polysaccharides pointed out some structure-activity relationships, with **93**, **95** and **96** carrying Fuc branches on GlcA units as well as **88** with trisulfated Fuc units on GalNAc residues showing a behavior similar to low molecular weight species obtained by partial depolymerization of natural fCSs [73]. Nonetheless, the random distribution of Fuc branches between positions 2 and 3 of GlcA units in **93**–**96** as well as between positions 4 and 6 of GalNAc residues in **88**–**91** hampered more detailed structure-activity relationship investigations. To overcome these limitations, a new chondroitin acceptor (**84**, Scheme 8), obtained by mild hydrolysis of benzylidene residues of polysaccharide **82** without a significant cleavage of glycosidic bonds, was very recently employed for the regioselective insertion of Fuc branches at GalNAc *O*-6 site [74]. Furthermore, the semi-synthesis of low molecular weight fCS polysaccharides with Fuc units exclusively linked at GlcA *O*-3 positions―as in most of natural fCSs―has been very recently accessed [78]. This result was achieved by applying the one-pot 3,6-lactonization/benzoylation/lactone methanolysis sequence―already developed on di-, tetra- and hexasaccharide chondroitin derivatives (Scheme 5 and Scheme 6)―also on microbial sourced CS-0 polysaccharide in order to obtain a polymeric acceptor with a single, free hydroxyl per repeating unit exclusively placed at GlcA *C*-3 site [79].

**Scheme 9:**
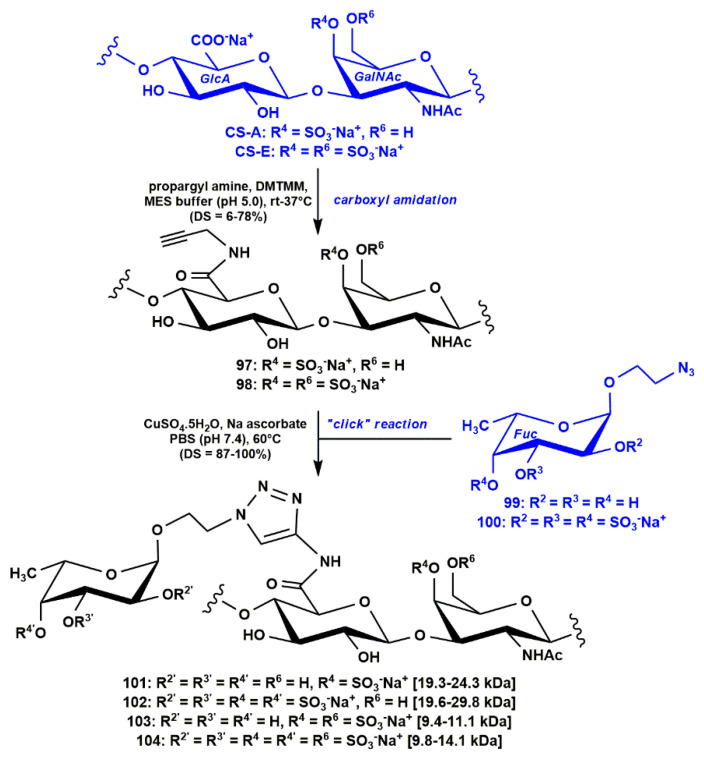
Semi-synthesis of polysaccharides with amide-linked Fuc branches from CS-A and CS-E [80]; abbreviations: 4-(4,6-dimethoxy-1,3,5-triazin-2-yl)-4-methylmorpholinium chloride (DMTMM), 4-morpholineethanesulfonic acid (MES), phosphate buffered saline (PBS).

A small library of low molecular weight polysaccharides resembling fCS structure, but with Fuc branches linked to the polymeric backbone through amide instead of glycosidic linkages, was prepared starting from two differently sulfated CS polysaccharides (CS-A and CS-E) [80]. The semi-synthesis relied upon a CuAAC “click” reaction between chondroitin polysaccharides derivatives **97** and **98** carrying *N*-propargyl amides with different degrees of substitution on GlcA units and fucosides **99** and **100** displaying a 2-azidoethyl aglycone (Scheme 9). The obtained grafted polysaccharides **101**–**104**, showing different sulfation patterns on Fuc and/or GalNAc units as well as a different degree of fucosylation, were assayed for their anticoagulant activity. Only polysaccharides **103** and **104** with the lowest degree of fucosylation (14%) clearly displayed an inhibition of the intrinsic coagulation pathway. This suggests a role of both GlcA carboxylic acid moieties and GalNAc sulfation degree for the anticoagulant activity of these fCS mimetics.

Semi-synthetic, low molecular weight polysaccharides with a non-natural fCS structure were also obtained by derivatization of fCSs themselves. In particular, a free-radical, partially depolymerized fCS from *Thelenata ananas* that shows 3-*O*-, 4-*O*- and 2,4-di-*O*-sulfated-Fuc branches [54], was derivatized through five different kinds of semi-synthetic modifications: random *O*-acylation (acetylation, propionylation or succinoylation) [81], GlcA carboxylic acid esterification (ethylation, benzylation or 1-butenylation) or reduction to alcohol, GalNAc *N*-deacetylation or Fuc de-branching [43]. All the derivatizations―except acylations with low degrees of substitution (up to 40%)―caused a reduction of the anticoagulant activity, with defucosylation with the greatest effect, as expected [10,11,12].

## 4. Conclusions and Perspectives

The highly promising activity of fCS polysaccharides extracted from sea cucumbers in blocking the intrinsic coagulation cascade has prompted several studies on this topic in the last decade. Reviews focused on structural diversity [9,18,19], characterization methods [17,23] and bioactivities [4] of fCSs have been reported, whereas to the best of our knowledge no review paper on (semi)-syntheses of (macro)molecules resembling the structure of natural fCS polysaccharides has appeared in the literature yet. Here we have filled this gap, by including in this review not only all the total synthetic and semi-synthetic strategies to fCS oligosaccharides and low molecular weight polysaccharides reported up to now, but also very recent achievements on semi-synthetic glycoclusters displaying multiple copies of fCS species. For all the targets, the total synthetic and/or semi-synthetic strategy has been discussed, underlining for each approach advantages and drawbacks and also reporting the main results on structure-bioactivity relationships.

Despite the several achievements obtained up to now by studies of fCS chemistry and biology, there are still some key points that should be addressed in a near future. Specifically, extensive structure-activity relationships of fCS have yet to be reported. Therefore, fast, easily scalable and/or cost effective (semi)-synthetic methods to fCS oligosaccharides, plausibly higher than an octasaccharide (the minimum structural unit able to confer a remarkable anticoagulant activity), should be developed. Furthermore, the chemical space around the natural fCS structure should be explored further and more intensively, in order to access non-natural oligosaccharides, low molecular weight polysaccharides or multivalent compounds that could display similar or even more powerful and interesting biological activities than all the fCS-related species investigated up to now. Therefore, we foresee that the research targeting the (semi)-synthesis of fCS oligo- and polysaccharides and analogs thereof will attract a growing interest in the next years and bring even more advances than in the last decade in the field of synthetic chemistry inspired to marine biomolecules.
